# Diabetes mellitus and drug abuse during pregnancy and the risk for orofacial clefts and related abnormalities**^1^**

**DOI:** 10.1590/1518-8345.0815.2701

**Published:** 2016-08-08

**Authors:** Ivy Kiemle Trindade-Suedam, Lília Maria von Kostrisch, Luiz André Freire Pimenta, Carlos Antônio Negrato, Solange Braga Franzolin, Alceu Sergio Trindade

**Affiliations:** 2 PhD, Associate Professor, Faculdade de Odontologia de Bauru, Universidade de São Paulo, Bauru, SP, Brazil.; 3 RN, Hospital de Messejana Dr. José Alberto Studart Gomes, Secretaria de Municipal de Saúde de Fortaleza, Fortaleza, CE, Brazil. Doctoral student, Hospital de Reabilitação de Anomalias Craniofaciais, Universidade de São Paulo, Bauru, SP, Brazil.; 4 Clinical Professor, School of Dentistry, University of North Carolina, Chapel Hill, NC, United States.; 5 PhD.; 6 PhD, Assistant Professor, Universidade Sagrado Coração, Bauru, SP, Brazil.; 7 PhD, Full Professor, Faculdade de Odontologia de Bauru, Universidade de São Paulo, Bauru, SP, Brazil.

**Keywords:** Cleft Palate, Diabetes Mellitus, Epidemiology, Substance-Related Disorders

## Abstract

**Objective:**

to assessed the prevalence of diabetes mellitus (DM) and drug abuse in mothers of children with orofacial clefts (OFC).

**Methods:**

325 women who had children (0-3y) with clefts were interviewed. Data regarding type of diabetes, use of legal/illegal drugs during pregnancy, waist girth and fasting blood sugar at the first prenatal consult were collected.

**Results:**

twenty seven percent of the women had DM, out of these, 89% had gestational DM, 5,5% type 1 DM and 5,5% type 2 DM. The prevalence of DM in mothers of children with OFC was 27%, it is significantly higher than the average Brazilian population which is 7.6% (p<0.01) (OR=4.5, 95%CI=3.5-5.8). Regarding drug abuse during pregnancy, 32% of the mothers used drugs and a significant positive correlation was observed between drug abuse and the occurrence of clefts and other craniofacial anomalies (p=0.028) (OR=2.87; 95%CI=1.1-7.4).

**Conclusions:**

DM and drug abuse during pregnancy increases the risk for OFC and related anomalies and early diagnosis of DM and prevention of drug abuse, especially in pregnant women, should be emphasized.

## Introduction

Diabetes mellitus (DM) is a metabolic disease resulting in hyperglycemia, either because of the low insulin levels or due to insulin resistance. According to the World Health Organization (WHO) definition, metabolic syndrome is significantly associated with age, physical activity, dyslipidemia, hypertension, treatment with oral antihyperglycemic medication, and HbA1c levels >7%^1^.

The global prevalence of DM for all ages was estimated to be 8,3% and is projected to almost double in 2035^2^, even in low and middle-income countries such as Brazil^3^. Data obtained by the Brazilian government show that the prevalence of DM in the adult population was 6.3%^4^.

Gestational diabetes mellitus (GDM) is defined as a carbohydrate intolerance initially diagnosed during pregnancy. Author^5^ stated that the pregnancies of women who were both obese and diabetic were 3 times as to result in an offspring with a craniofacial defect than were those of nonobese, nondiabetic women, suggesting that obesity and diabetes mellitus contributes in the pathogenesis of congenital anomalies.

The assumption that GMD is associated with increased occurrence of syndromes and malformations might be attributed to the deleterious effect of hyperglycemia in the early stage of pregnancy. This indicates that poor glycemic control during pregnancy increases the risk of congenital defects^6^. However, it is still controversial as to whether --- severe levels of hyperglycemia are associated with higher risk of adverse events during pregnancy.

As well as hyperglycemia, drug abuse (DU) during pregnancy represents a high risk behavior for the occurrence of several congenital malformations including orofacial clefts^7^ and represents one of the most significant social problems around the world^8^.

Congenital anomalies are extremely variable regarding the type as well as the causes. Orofacial clefts (OFC) are functional and structural malformations as a result of an abnormal development of the maxillary complex during embryogenesis and can be characterized by the lack of continuity of the upper lips, upper alveolar ridge and palate and can affect these structures partially or completely^9^. 

The etiology of OFC is complex and multifactorial. Genetic factors, environmental factors and the interaction of both may interfere with the intrinsic mechanism of pregnancy causing embryological abnormalities. Mutations in IRF6 , MSX1, FOXE1, MTHFR C677T, FAF1 and TGFB genes represent the genetic factors^10^ ^-^ ^12^. Among the environmental factors are maternal nutritional status (hypo- and hypervitaminosis), smoking and alcohol consumption during pregnancy, occupational exposures to chemicals (solvents and pesticides), exposure to X-rays, maternal illness during pregnancy including diabetes mellitus, epilepsy and viral infections, and the inadvertent use of some medications such as benzodiazepines and corticosteroids^13^ ^-^ ^16^.

DM have shown that it represents a potential etiological factor for several anomalies, indicating that women with diabetes present higher chances of having children with congenital anomalies, including OFC^14^. However, no data from the Brazilian population was found in the literature. 

The Hospital for Rehabilitation of Craniofacial Anomalies is a Brazilian public Hospital located in the centre of the São Paulo State and is recognized by the World Health Organization as a world reference center for the treatment of cleft lip and palate and related anomalies. HRAC is a tertiary hospital and the fundings for the treatment of the 100.000 patients registered come from the University of São Paulo and from the Brazilian health public system. People from all over Brazil can be registred at HRAC, independently of their social status. Therefore, this study assessed the prevalence of DM and DU in mothers of children born with OFC in the Hospital for Rehabilitation of Craniofacial Anomalies - University of São Paulo (HRAC/USP), Brazil, and compared it with that of a background Brazilian population.

## Method

This study was approved by the IRB of HRAC/USP. The sample size for this study was calculated based on data showing that the prevalence of diabetes in Brazil is between 6% and 8%^3^ ^-^ ^4^ and a number of 324 individuals was reached. 

This was a cross-sectional observational study, with data collected during a time interval of 12 months, until a number of 325 women and their offspring born cleft lip and palate (CLP) was reached. Participants were recruited as follows: the interviewer was introduced to the mothers by the physician responsible for the first appointment or by one of the nurses that comprise the multidisciplinary team of the HRAC/USP. The purposes of the study were explained to them and only mothers who agreed to participate and signed the informed consent form were enrolled in the study. Considering that the HRAC/USP is a hospital for the exclusive treatment of individuals with CLP, all women evaluated in the present study have had children with CLP.

Data was collected on a private room by one the authors of the present study, a nurse trained for the application of the questionnaire. All children were followed up at HRAC/USP. Mothers were examined and the results from the fasting glucose test (level of glucose during pregnancy expressed in mg/dL) were collected by recording data from the first pre-natal exam. Abdominal circumference was assessed and measures >80cm were considered as an indicative of obesity^17^. In addition to the clinical data, a questionnaire with 24 questions was given to the mothers and they answered questions regarding the type of diabetes (Type 1, Type 2 or Gestational) and any other comorbidities associated with the disease. Women were also asked to answer questions regarding history of hypertension, obesity, use of legal and illegal drugs during pregnancy, and any lists of medication. Drug categorization into licit or illicit followed the United Nations Office on Drugs and Crime - UNODC classification (2015), which states that the term illicit drugs describes drugs which are under international control (and which may or may not have licit medical purposes) but which are produced, trafficked and/or consumed illicitly. Among the most consumed licit use drugs worldwide are alcohol and tobacco, while cannabis, cocaine and crack represent the most common illicit use drugs^18^.

Data regarding age, race, educational level and symptoms related to DM were also collected. Educational level was ranked according to The International Standard Classification of Education , ISCED - 2011, from the United Nations Educational, Scientific and Cultural Organization - UNESCO^19^, as follows: 1) primary education (fundamental skills in reading, writing and mathematics), 2) lower secondary education (based on primary education, with a more subject-oriented curriculum), 3) upper secondary education (final stage of secondary education preparing for tertiary education and/or providing skills relevant to employment), 4) post-secondary non-tertiary education (learning experiences that prepare for labour market entry and/or tertiary education), and, 5) bachelor / tertiary education (programmes designed to provide intermediate academic and/or professional knowledge, skills and competencies leading to a first tertiary degree or equivalent qualification).

Proportions were compared by calculating the rate difference and its 95% CI (confidence interval). One way Analysis of Variance test and the Student´s t test were used to assess the possible effects of glucose level and maternal age in determining different types of clefts and related anomalies, respectively. Chi-Square test and Fisher exact test were used to determine the significance of the association between the use of licit and illicit drugs and the type of cleft and related anomalies, respectively. Results were assessed by the *Statistica software*. A p value of < 0.05 was considered statistically significant.

## Results

The mothers ranged in age from 15 to 50 years old, with a mean age of 29 years old, while the children ranged in age from 0 to 3 years. The majority of women had completed middle school 155 (48%). Regarding race, 177 (55%) self-reported as white and 141 (43%) as black or of Afro-descendant. Regarding clinical symptoms of DM, 165 (51%) of the mothers reported feeling tired and lethargic early in the morning, 143 (44%) reported postpartum weight loss and 140 (43%) reported asthenia, as seen on Table 1.

**Table 1:** Distribution of the women population regarding age, race, educational level and symptoms related to diabetes mellitus (DM). Bauru-SP, Brasil, 2012.

Variables		N	%
AGE			
	15 - 20	36	11.1
	21 - 30	157	48.3
	31 - 40	117	36.0
	41 - 50	15	4.6
RACE*			
	WHITE	177	54.5
	AFRICAN-DECENT	111	34.2
	BLACK	30	9.2
	ASIAN	04	1.2
	NATIVE INDIAN	03	0.9
EDUCATIONAL LEVEL†			
	ILLITERATE	02	0.6
	PRIMARY EDUCATION	114	35.1
	LOWER SECONDARY EDUCATION	155	47.7
	UPPER SECONDARY EDUCATION	52	16.0
	TERTIARY EDUCATION	02	0.6
SYMPTOMS OF DM‡			
	WEIGHT LOSS	143	12.5
	ASTHENIA	140	12.2
	POLYPHAGIA	124	10.8
	POLYDIPSIA	123	10.7
	POLYURIA	95	8.3
TOTAL (N)		325	100

* Criteria adopted by IBGE for race (Brazilian Government);

†The International Standard Classification of Education (ISCED) - 2011, from the United Nations Educational, Scientific and Cultural Organization (UNESCO); ‡DM: diabetes mellitus

From the 325 women who answered the questionnaire, 28 came to the HRAC hospital already with the diagnosis of DM. From the remaining 297 women, 60 presented with pre-natal glycemic levels ≥ 92 mg/dL at the first consult with the obstetrician. Therefore, following the criteria of the American Diabetes Association and the International Diabetes Federation, they were added to the initial 28 women with a previous diagnosis of DM, resulting in 88 women with DM (27%). Out of these 88 women, 78 were diagnosed with GDM (89%), 5 with type 1 (5,5%) and 5 with type 2 DM (5,5%) (Table 2). This finding is significantly higher than the average Brazilian population which is around 7%^(3-4)^ (p<0.01).

**Table 2:** Distribution of women who gave birth to children with OFC in the study. Bauru-SP, Brasil, 2012.

Diagnostic		n	%	95% Confidence Interval including continuity correction	
DM*		88†	27	22.39	32.07
	GDM‡	78	24	19.50	28.81
	DM1§	5	1.5	0.29	3.09
	DM2||	5	1.5	0.29	3.09

* DM: diabetes mellitus; †*(p<0,01) in relation to the general population (prevalence of Brazilian women with DM),* ‡GDM: gestational diabetes mellitus, §DM1: type 1 diabetes mellitus; ||DM2: type 2 diabetes mellitus

**Figure 1:**
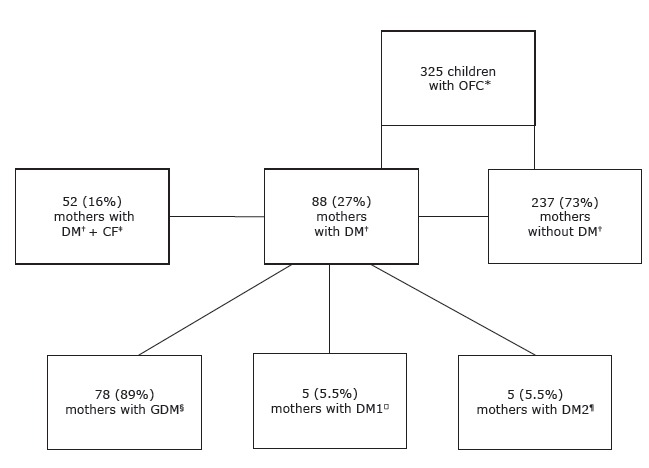
Process of identification of mothers with Diabetes Mellitus

From the total of 88 women with a diagnosis of DM, all factors that could contribute to the development of congenital anomalies such as consumption of alcohol, smoking, use of illegal drugs or potential teratogenic medications as well as patients with obesity, high blood pressure or dyslipidemia were excluded. This resulted in 52 women (16%) being diagnosed with maternal hyperglycemia as the isolated causal factor for congenital anomalies, including orofacial cleft (Figure 1).

* OFC: orofacial clefts; †DM: diabetes mellitus; ‡CF: confounding factors (alcohol consumption, smoking or use of medications or legal/ illegal drugs during pregnancy, obesity and high blood pressure); § GDM: gestational diabetes mellitus, ǁDM1: type 1 diabetes mellitus; ¶DM2: type 2 diabetes mellitus.

The odds ratio of hyperglycemic mothers including confounding factors/global prevalence of DM was 4.5 CI (3.5 to 5.8) (n=88) and the odds ratio of hyperglycemic mothers excluding confounding factors/global prevalence of DM was 2.3 CI (1.7 to 3.1) (n=52). 

The level of glucose and maternal age during pregnancy and their relations with the type of cleft and related anomalies are presented in Table 3. It can be seen that the greater the level of glucose, the severe is the type of cleft. For example, mothers who gave birth to children with CLP had a mean level of glucose of 169mg/dL while mothers who gave birth to children with cleft lip had a mean level of glucose of 117mg/dL However, no significant differences were observed. It was also observed that increasing age is associated with the severity of the cleft type and with the presence of related anomalies. In other words, older mothers gave birth to children with more severe clefts and with related anomalies associated to CLP, such as Pierre Robin sequence, hand and feet malformations, hydrocephalus and Down syndrome, among others. Yet again, no significant differences were observed.

**Table 3:** Level of glucose during pregnancy and maternal age and their relations with the type of cleft and related anomalies. Bauru, SP, Brasil, 2012.

Level of glucose during pregnancy (mg/dL)			Maternal age during pregnancy	
			N	x±sd
Type of cleft	CL*	117.44±20.34	9	30.11±7.25
	iCP**^†^**	143.33±64.63	33	21.18±6.42
	CLP**^‡^**	169.27±126.79	45	30.33±6.32
Related anomalies	Presence	160.39±119.95	44	31.00±6.38
	Abscence	147.61±76.88	43	30,26±6.45

* CL: cleft lip; †iCP: isolated cleft palate; ‡CLP: cleft lip and palate / *no significant differences observed;*

Results showed that 28 women (32%) of the present sample used drugs during pregnancy. From these, 64% of their offspring had complete CLP, i.e, the most severe type of cleft. This number decreased to 46% in mothers who have not used drugs during pregnancy. However, differences were not statistically significant. Moreover, a particular data stands out, 69% of the offspring of mothers who used drugs during pregnancy was born with CLP associated to other craniofacial anomalies while only 42% of the children was born with the same characteristics from mothers who haven't used drugs during pregnancy (Table 4). This difference was statistically significant (p=0.028). It was also observed an increased odds of having a child with CLP and with related anomalies among women who used drugs during pregnancy (OR=2.87; 95% CI=1.1-7.4).

**Table 4:** Licit and illicit drug abuse during pregnancy and its relation with the type of cleft and related anomalies. Bauru-SP, Brasil, 2012.

		Drug abuse in pregnacy		Licit drug in Pregnancy			Illicit drug in pregnacy
		Yes	No	Yes	No	Yes	No
		n(%)		n(%)			n(%)
Type of cleft	CL*	2(7%)	7(12%)				
	iCP†	8(29%)	25(42%)				
	CLP‡	18(64%)	27(46%)				
Related anomalies	presence	20(69%)§	25(42%)	19(68%)||	26(43%)	3(75%)	41(49%)
	abscence	9(31%)	34(58%)	9(32%)	34(57%)	1(25%)	42(51%)

* CL: cleft lip; †iCP: isolated cleft palate; ‡CLP: cleft lip and palate §*p=0.028;* ||*p=0.030*

Considering this important data regarding the use of drugs and the occurrence of other craniofacial anomalies associated to CLP, an attempt to differentiate the effects of licit and illicit drugs in the fetus was done (Table 4). Results have shown that there is a positive correlation between the use licit drugs in the periconceptional period and the occurrence of related craniofacial anomalies (p=0.03). This correlation was not observed for the illicit drugs

## Discussion

The current study shows that women with diabetes mellitus are more likely to have children with orofacial clefts when compared to women without DM. The prevalence of DM in the sample analyzed was 27% while on the global and in the Brazilian populations this percentage is significantly lower, of around 7%^3^ ^-^ ^4^. The study also shows that drug abuse during pregnancy increases in almost 3 times the risk for the occurrence of orofacial clefts associated with other craniofacial anomalies. 

Due to the high prevalence of DM in this population, the comorbidities associated with congenital anomalies were excluded to assess exclusively maternal hyperglycemia as a possible causal factor of OFC. Thus, factors such as consumption of legal or illegal drugs during the gestational term were excluded^20^ ^-^ ^21^. Women with abdominal circumferences >80cm were considered obese and were not included in the second analysis^17^. Hypertension and dyslipidemia were additionally excluded, because these clinical conditions are commonly associated with diabetes and represent risk factors for the development of metabolic syndrome^22^ ^-^ ^23^. Women that were treated with antibiotics, anti-hypertensive, anti-emetics, non-steroid anti-inflammatories, anticonvulsant, corticoids and other types of analgesics were also excluded from the analysis since the use of these medications during pregnancy could be a factor for the development of OFC^15^ ^-^ ^16^. It is important to mention that the majority of women reported that they have had supplements of folic acid (58%), iron (59%), and multi-vitamins (23%) during the gestational period for prevention of congenital anomalies.

Therefore, when the comorbidities during pregnancy previously mentioned were excluded, the prevalence of DM dropped to 16%, however it still represents more than twice the percentage of DM in the global population. In other words, it is possible to infer that hyperglycemia during pregnancy increases the risk for the occurrence of OFC. These results are in accordance with the findings of other study^24^ that have mentioned that maternal diabetes can induce congenital malformations in laboratory animals and in humans, including facial deformities and defects in neural tube closure. These authors have also stated that the incidence of birth defects in newborns of women with diabetes is approximately 3-5 times higher than among women without diabetes.

The findings presented in this study reinforce the need for rigorous control of DM during the gestational period. Among women with DM (n=88) from this sample, 60 (68%) did not have any control of glycemic levels during gestation, suggesting that these fetuses were exposed to maternal hyperglycemia during embryogenesis. This poor glycemic control is probably due to the low social status of this population. Regarding their educational level, the majority of them completed middle school and in some cases, they reported that the birth of a child with a congenital anomaly forced them to stop studying to take care of the child. There are also reports from those mothers of anxiety and depression when they were surprised with the information that their children were diagnosed with some type of congenital anomalie^25^.

The authors believe that the number of women with diabetes could be even higher, since the information about the glycemic test was not conducted prospectively as part of the study, and there is no information about the conditions in which the glycemic test was performed. Many of the women on this study live far from the centers where the blood was drawn. Ideally, the glycemic test should be performed after a fasting period of at least 8 hours and no longer than 14 hours. 

The primary objective of this study was to investigate the prevalence of diabetes mellitus in mothers of children with CLP. However, during data collection, an important finding has emerged. Among the group of mothers who referred using drugs during pregnancy, 69% of children were born with orofacial clefts and with other congenital anomaly, whereas in the group of mothers who did not use drugs, the percentage of children with orofacial clefts associated with other anomalies was significantly lower (42%). This means that mothers who use drugs during pregnancy have nearly 3 times more likely to have a child with CLP associated with other congenital anomalies than the mothers from this sample who have not used drugs during pregnancy. Pierre Robin Sequence (9%) was the most prevalent congenital malformation observed, followed by cardiopathies (5%), foot and hand malformation (3%), hearing issues (1%) and other syndromes. 

Regarding the type of drug abused, it has been shown that the use of licit drugs such as alcohol, tobacco, benzodiazepines, stimulants, anticonvulsants and other antidepressants increase the risk for the occurrence of other congenital anomalies together with CLP. This positive correlation could not be observed for the illicit drugs, such as cocaine and cannabis, probably because of the small sample size. It is important to mention that the concern of assuming an illegal behavior may have underestimated the number of mothers who used drugs during pregnancy.

The results of this study highlight that prevention campaigns to drug abuse should also focus on the pregnant population whose condition, unfortunately, do not keep them free from the use of licit or illicit chemical substances during pregnancy. The study also highlights the importance of glycemic control for early detection of hyperglycemia, even before conception and especially in pregnant women. If not detected and treated, hyperglycemia could lead to congenital anomalies. DM and DU could also result in physical, psychosocial and economic problems for families and society as well as increasing costs for the health care system.

More prospective studies should be conducted to evaluate the prevalence of DM and DU in mothers of children born with OFC. Thus, the association between the severity of the DM, its relationship to the gestational term, and the type of clefts observed could be studied. Ideally, the focus should be in preventing these congenital anomalies through early diagnosis of DM and prevention of DU. In addition, a multidisciplinary approach should be developed, which could lead to more comprehensive care and could minimize the development of different congenital anomalies.

## Conclusion

It can be concluded that hyperglycemia and drug abuse during pregnancy increases the risk for occurrence of OFC and related anomalies and, consequently, early diagnosis of DM and prevention of DU, especially in pregnant women, should be emphasized.
